# Morphological and functional cardiac consequences of rapid hypertension treatment: a cohort study

**DOI:** 10.1186/s12968-021-00805-5

**Published:** 2021-10-25

**Authors:** Andrew N. Jordan, Jon Fulford, Kim Gooding, Christine Anning, Lindsay Wilkes, Claire Ball, Nicola Pamphilon, David Mawson, Christopher E. Clark, Angela C. Shore, Andrew S. P. Sharp, Nicholas G. Bellenger

**Affiliations:** 1 Vascular Medicine, NIHR Exeter Clinical Research Facility, Exeter, UK; 2 Institute of Biomedical and Clinical Science, University of Exeter Medical School, Exeter, EX2 5AX UK; 3 Primary Care Research Group, Exeter College of Medicine and Health, Smeall Building, St Luke’s Campus, Magdalen Road, Exeter, EX1 2LU UK; 4 Department of Cardiology, Royal Devon and Exeter Hospital, Exeter, UK; 5 Diabetes and Vascular Research Centre, Royal Devon and Exeter NHS Foundation Trust, Barrack Road, Exeter, EX2 5DW UK

**Keywords:** Left ventricular hypertrophy, Feature tracking, Strain, Torsion, Rapid treatment, Hypertension

## Abstract

**Background:**

Left ventricular (LV) hypertrophy (LVH) in uncontrolled hypertension is an independent predictor of mortality, though its regression with treatment improves outcomes. Retrospective data suggest that early control of hypertension provides a prognostic advantage and this strategy is included in the 2018 European guidelines, which recommend treating grade II/III hypertension to target blood pressure (BP) within 3 months. The earliest LVH regression to date was demonstrated by echocardiography at 24 weeks. The effect of a rapid guideline-based treatment protocol on LV remodelling, with very early BP control by 18 weeks remains controversial and previously unreported. We aimed to determine whether such rapid hypertension treatment is associated with improvements in LV structure and function through paired cardiovascular magnetic resonance (CMR) scanning at baseline and 18 weeks, utilising CMR mass and feature tracking analysis.

**Methods:**

We recruited participants with never-treated grade II/III hypertension, initiating a guideline-based treatment protocol which aimed to achieve BP control within 18 weeks. CMR and feature tracking were used to assess myocardial morphology and function immediately before and after treatment.

**Results:**

We acquired complete pre- and 18-week post-treatment data for 41 participants. During the interval, LV mass index reduced significantly (43.5 ± 9.8 to 37.6 ± 8.3 g/m^2^, p < 0.001) following treatment, accompanied by reductions in LV ejection fraction (65.6 ± 6.8 to 63.4 ± 7.1%, p = 0.03), global radial strain (46.1 ± 9.7 to 39.1 ± 10.9, p < 0.001), mid-circumferential strain (− 20.8 ± 4.9 to − 19.1 ± 3.7, p = 0.02), apical circumferential strain (− 26.0 ± 5.3 to − 23.4 ± 4.2, p = 0.003) and apical rotation (9.8 ± 5.0 to 7.5 ± 4.5, p = 0.003).

**Conclusions:**

LVH regresses following just 18 weeks of intensive antihypertensive treatment in subjects with newly-diagnosed grade II/III hypertension. This is accompanied by potentially advantageous functional changes within the myocardium and supports the hypothesis that rapid treatment of hypertension could improve clinical outcomes.

*Trial registration*: ISRCTN registry number: 57475376 (assigned 25/06/2015).

## Background

Hypertension is associated with disruption of the structure and function of the left ventricular (LV) myocardium. Foremost amongst these changes is LV hypertrophy (LVH), quantified as LV mass (LVM) and normalised to body surface area (BSA) as LVM index.

Increased LVM has been shown to be an independent predictor of mortality in the Framingham cohort [1]. Further cohort studies have demonstrated that LVM also stratifies risk in subjects with and without coronary artery disease [2] and specifically in the hypertensive population [3].

Following treatment of hypertension, persistent LVH is an indicator of poor prognosis, whereas complete regression almost normalises cardiovascular risk [4]. The association of treatment-induced LVM regression with favourable prognosis is independent of baseline LVM and degree of blood pressure (BP) reduction [5]. As a measure of end-organ damage, LVM may be more closely linked to prognosis than office BP reductions [6], though low event rates in cohort studies of uncomplicated essential hypertension hamper definitive conclusions as to the relative weighting of these associations [4–7]. The timing of LVH regression is also unclear, with the most rapid improvement demonstrated in a longitudinal study of 24 weeks’ duration using echocardiography to quantify LVM before and after treatment for patients enrolled with grade I or II hypertension (mean baseline office BP: 164/93mmHg) and receiving either standard BP management or intensive management within the study [8].

**Fig. 1:**
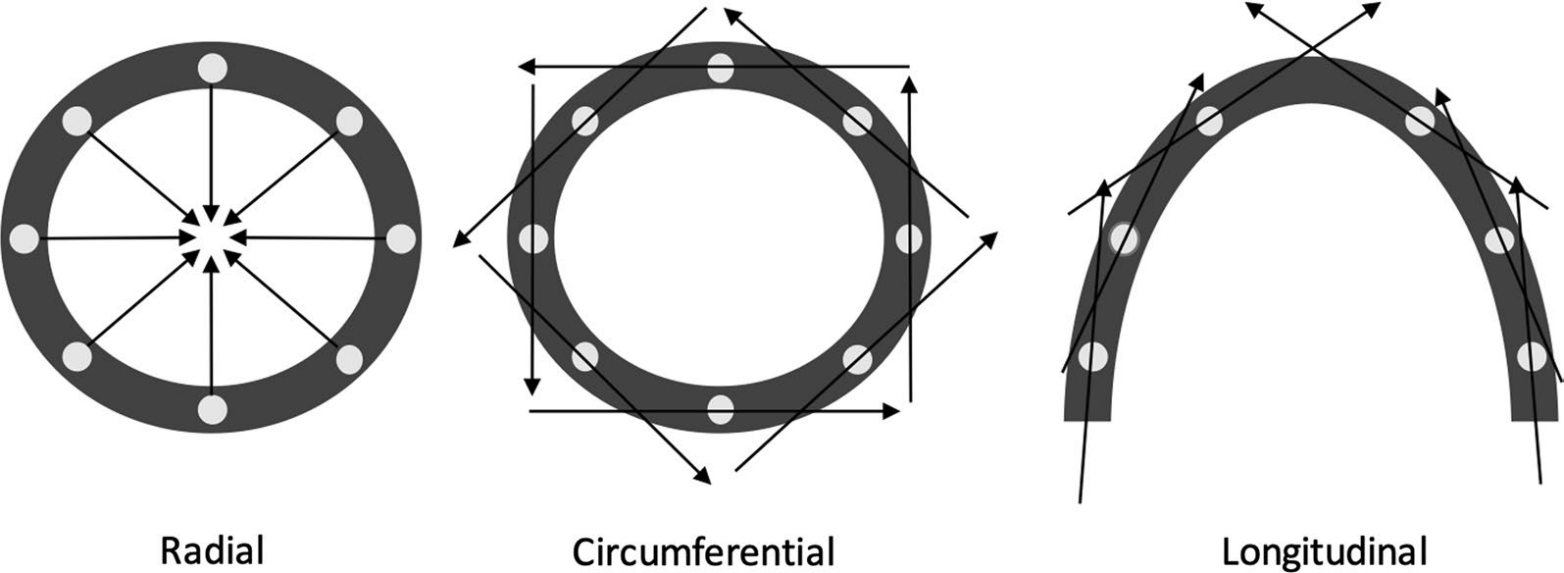
Schematic representation of strain parameters in relation to the LV myocardium

LVH in hypertension allows the heart to maintain a normal, or even supra-normal LV ejection fraction (LVEF) in the face of increased afterload [9], at least in the early stages of the disease. However, despite a normal LVEF, systolic function is demonstrably not normal in hearts of hypertensive patients. Cross-sectional echocardiographic studies using speckle tracking have shown a reduction in measures of myocardial strain (deformation per unit length) in hypertensive individuals versus normotensive control subjects [10–12], which can be described according to the direction of deformation relative to the cardiac axis (Fig. 1). In individuals with hypertension, speckle tracking has demonstrated a reduction in myocardial global longitudinal strain (GLS) and global radial strain (GRS) [10, 11]. Analysis of circumferential strain magnitude in hypertensive subjects have been inconsistent, with some studies suggesting a reduction in global circumferential strain (GCS) in hypertension [10] and others determining no significant difference compared with normotensive controls [11, 12].

LV torsion, defined as the relative systolic twisting motion as a consequence of basal clockwise rotation and apical anticlockwise rotation normalised to LV length [13], has been shown to increase in amplitude in hypertensive subjects when measured using echocardiographic speckle tracking [10] and cardiovascular magnetic resonance (CMR) imaging [14]. This may, at least in part, facilitate the preservation of LVEF seen in hypertensive subjects despite the reduction in longitudinal and radial strain [14].

Resolution of LV systolic dysfunction, defined according to GLS, GCS and GRS, in subjects with treated hypertension when compared with untreated hypertension and normotensive controls has been demonstrated in a cross-sectional study [10]. Longitudinal studies investigating the effect of antihypertensive treatment on LV myocardial mechanics have concentrated on circumferential and longitudinal strain [15, 16] or longitudinal strain alone [8]. The effects of antihypertensive treatment on radial strain and LV torsion have not been reported in longitudinal studies. Furthermore, the shortest duration of antihypertensive treatment shown to improve longitudinal and circumferential strain is 24 weeks, within the study design described above and including a heterogenous cohort of participants treated either with intensive management or standard care [8].

CMR has been shown to have greater accuracy in the measurement of LVM when compared with 2D echocardiography [17]. CMR feature tracking for strain assessment, which uses post-processing technology to track intramyocardial features throughout the cardiac cycle [18], has been shown to be more reproducible than older CMR techniques [19].

This study therefore aims to use CMR to establish for the first time whether LVH regression occurs within 18 weeks of commencing antihypertensive treatment in individuals with never-treated essential hypertension. Furthermore, CMR feature tracking will supplement this through a comprehensive assessment of myocardial strain in all planes relative to the cardiac axis: the first time that this technology has been applied in a longitudinal study, determining the cardiac effects of antihypertensive treatment with greater accuracy than previous studies using echocardiography.

## Methods

Potential participants aged 18–79 years and never treated for hypertension were identified by their usual care clinician following an office systolic BP of ≥170mmHg. Ambulatory monitoring was then used to confirm at least grade II hypertension (daytime average systolic BP ≥150 mmHg) prior to enrolment.

Exclusion criteria were: renal impairment (eGFR < 60 ml/min/1.73 m^2^, Hemoglobin <10 g/dl, platelet count <100 × 10^9^/l or bleeding diathesis, pregnancy or breastfeeding, inability to provide informed consent, hypertension-related event (including stroke or acute kidney injury) within the preceding 3 months, or any condition, including hypertensive urgency, requiring immediate BP lowering or tailored antihypertensive strategy.

Participants underwent a treatment programme involving fortnightly nurse-led consultations. At each consultation stepwise intensification of antihypertensive treatment or further investigation was mandated for those not at office BP target according to a pre-defined protocol, as detailed previously [20]. Treatment targets and antihypertensive medication followed national and international consensus guidelines [21, 22].

CMR studies were undertaken immediately before and after 18 weeks’ antihypertensive treatment. Imaging was performed at the Exeter Magnetic Resonance Research Centre, St Luke’s Campus, University of Exeter using a 1.5T CMR system (Intera, Philips Healthcare, Best, Netherlands) with a 5-channel surface phased array coil. Our standard clinical hypertension CMR protocol was undertaken which includes 4-chamber, 2-chamber, 3-chamber and short axis stack balanced steady-state free precession cine imaging (balanced fast field gradient echo sequences, repetition time 3.2 ms, echo time 1.6 ms, 20–30 phases, slice thickness 8 mm, 1.2 × 1.2 mm spatial resolution). T1-weighted gradient echo sequences were used to determine renal and adrenal anatomy, followed by aortic and renal artery delineation using early gadolinium enhancement (0.15 mmol/kg gadopentate dimeglumine, Gadovist®, Bayer Healthcare Berlin, Germany), (repetition time 5.2 ms, echo time 1.5 ms, 40 slices, 8 mm slice thickness, 0.7 × 0.7 mm spatial resolution). Late gadolinium enhancement (LGE) gradient echo phase-sensitive inversion recovery sequences performed 8–10 min after gadolinium administration were acquired in three short axis slices and a long axis plane to assess for myocardial fibrosis or infiltration before and after antihypertensive treatment (repetition time 5.4 ms, echo time 2.6 ms, 8 mm slice thickness, 1.2 × 1.2 mm spatial resolution). This ensured that it would be possible to exclude other causes of LVH, such as Anderson-Fabry disease and amyloidosis, together with prior myocardial infarction, which could affect the volumetric and feature tracking analysis.

Post-hoc CMR data analysis was conducted by a single operator. LV volumes and LVM were calculated through planimetry of end-diastolic epicardial areas and end-diastolic and end-systolic endocardial areas for each short axis slice covering the entire left ventricle from mitral valve to apex, in line with standard protocols [23] and using commercially-available software (Extended MR WorkSpace, Philips Healthcare). Left atrial (LA) volume was determined using the biplane area length method [24]. LV long-axis length was assessed in a 4-chamber view by measuring the distance from the middle of the atrioventricular ring to the apex at end-diastole. Short-axis length was assessed in the same view at the papillary muscle insertion point. LV sphericity index was defined as the ratio of LV short axis length to long axis length and LV thickness was measured in the short axis at papillary muscle level in end-diastole.

Feature tracking analysis required dedicated software (TomTec Imaging Systems, 2-dimensional CPA MR, Cardiac Performance Analysis, Unterschleissheim, Germany). Short axis basal, mid-ventricular and apical image plane selection was determined as previously described [25]. LV endocardial and epicardial borders were manually traced with the initial contour set in end-diastole. The software then automatically traced the tissue voxels throughout the cardiac cycle, which was reviewed and repeated if myocardial tracking was not adequate visually.

Basal clockwise rotation (*ϕ*_*base*_), apical anticlockwise rotation *(ϕ*_*apex*_) and the distance between the basal and apical imaging planes (D) were used to derive twist and torsion:

$${Twist= {\phi}_{apex}- }\phi_{base}$$

$$Torsion= \frac{{\phi}_{apex}- {\phi}_{base}}{D}$$

### Study endpoints

The aim of the CMR investigation was to determine whether LVM index changed following 18 weeks of antihypertensive treatment.

Key further endpoints were the change in LV volume, LVEF, sphericity index, LA volume and strain parameters with treatment. The presence and distribution of LGE in our cohort was also evaluated, together with the incidence of detection of secondary causes of hypertension.

### Sample size and statistical analysis

A previous study assessing the change in CMR-defined LVM index with antihypertensive treatment found a mean reduction from 80.3 ± 15.7 to 70.1 ± 16.7 g/m^2^ after 52 weeks of treatment with a combination of an angiotensin converting enzyme inhibitor and calcium channel blocker [26]. An effect size of at least a 0.65 standard deviation difference in LVM index before and after antihypertensive treatment was therefore used to inform our study sample size given our study has a shorter intervention period but uses a technique better able to detect smaller changes in LVM.

We planned to enroll 50 participants into a clinical study of the feasibility and safety of rapid treatment of grade II/III hypertension [20]. Of these, 75% recruitment to paired CMR studies was anticipated. Using a two-tailed paired t-test (α = 0.05) comparison, 38 participants would enable the detection of a 0.55 SD with 90% power at a level of significance of 5%.

Statistical analysis was performed using STATA (version 14.1, StataCorp, College Station, Texas, USA). Baseline and outcome data are presented as means (± standard deviation) or medians (interquartile range) for continuous data depending on the normality of the data and counts (percentages) for categorical and binary variables.

Parametric data were analysed using a paired t-test, non-parametric data were analysed using Wilcoxon’s signed ranks test and proportions using a one-sample test of proportions. A two-sided *P* value threshold <0.05 was considered statistically significant. Univariate linear regression models were employed to determine the relationship between BP response during the study and markers of change in LV structure and function as outcome variables.

## Results

**Fig. 2:**
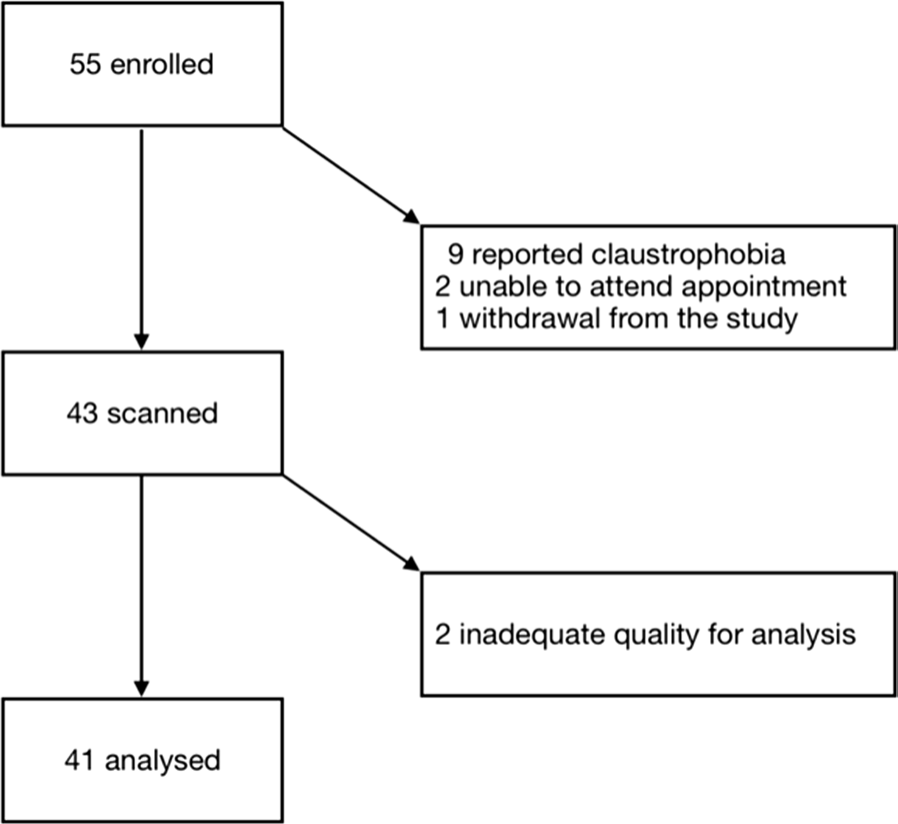
Number of enrolled participants in the clinical study of rapid hypertension treatment with MR imaging appropriate for analysis

Of the 55 participants recruited to the study, one did not complete the treatment protocol and nine did not complete 2 CMR studies due to claustrophobia. For two additional subjects, CMR data were subject to artefact and two patients were unable to attend their final appointments. The following results therefore refer to the remaining 41 participants (Fig. 2).

Median age of the analysis group was 59 years, 61% were male, there were no diabetic subjects and mean office BP reduced from 175/103 to 132/80 mmHg with treatment. Further characteristics prior to and following the treatment programme are summarised (Table 1).

**Table 1:** Characteristics of participants before and after 18 weeks’ antihypertensive treatment

Variable	Before treatment	After treatment	P value
Office systolic BP (mmHg)	174 ± 15.7	132 ± 11.9	< 0.001
Office diastolic BP (mmHg)	103 ± 9.3	80 ± 8.4	< 0.001
Daytime average systolic BP (mmHg)	163 ± 10.4	134 ± 10.4	< 0.001
Daytime average diastolic BP (mmHg)	93 ± 8.9	78 ± 6.8	< 0.001
Heart rate (bpm)	71 ± 12.0	64 ± 10.1	< 0.001^a^
BMI (kg/m^2^)	29.7 ± 5.3	29.7 ± 4.9	0.9
Current smoker (n)	5 (12%)	5 (12%)	1.0^b^
Alcohol (units/week)	8 (1–18)	9 (1–15)	0.8^a^
Fasting total cholesterol (mmol/L)	5.6 ± 1.2	5.6 ± 1.3	0.7
Creatinine (μmol/L)	75 ± 13.0	78 ± 13.4	0.3
HbA1c (mmol/mol)	38 ± 0.5	38 ± 0.6	0.6
Angiotensin receptor blocker (n)	0	37 (90%)	n/a
Calcium channel blocker (n)	0	40 (98%)	n/a
Thiazide diuretic (n)	0	24 (59%)	n/a
Aldosterone antagonist (n)	0	9 (21%)	n/a
α-blocker (n)	0	1 (2%)	n/a
β-blocker (n)	0	3 (7%)	n/a
Number of anti-hypertensives (n)	0	3 (2–3)	n/a

Expressed as mean ± standard deviation or median and interquartile range

*BMI* body mass index, *BP* blood pressure, *HbA1c* haemoglobin A1c

^a^Wilcoxon’s signed ranks test

^b^One-sample test of proportions

### Left ventricular mass and mass index

**Fig. 3:**
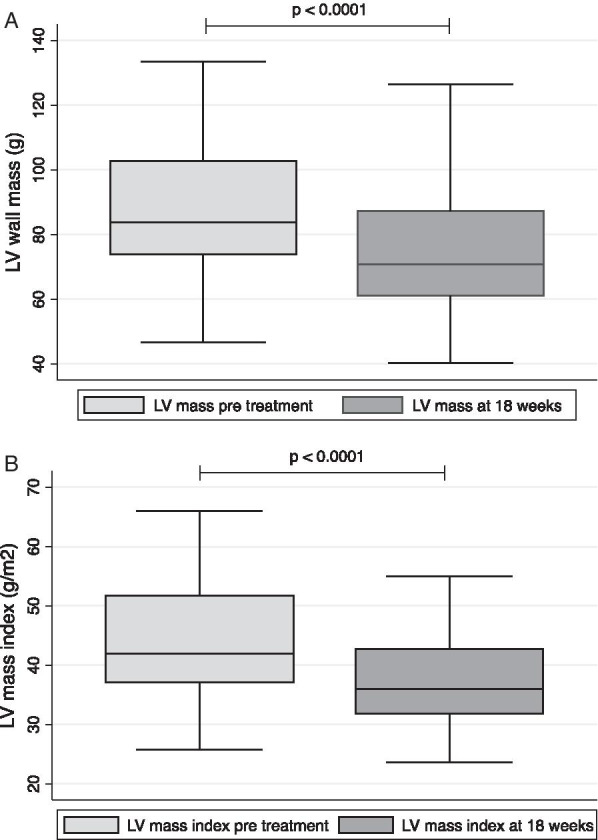
Left ventricular (LV) mass (**A**) and mass index (**B**) before and after 18 weeks’ antihypertensive treatment

LVM index reduced significantly after 18 weeks of antihypertensive treatment (43.5 ± 9.8 to 37.6 ± 8.3g/m^2^, p < 0.001). This marked reduction was also observed in non-indexed LVM (88.9 ± 24.5 to 76.8 ± 21.7 g, p < 0.001) (Fig. 3) and LV diastolic thickness (12.2 ± 2.0 to 10.5 ±1.6 mm, p < 0.001).

Change in LVM index was associated with diastolic BP change (β = 0.4, p = 0.01), and with systolic BP change (β = 0.3, p = 0.04).

Likewise, non-indexed LVM regression was associated with office diastolic BP change after 18 weeks of antihypertensive treatment (β = 0.4, p = 0.01). Non-indexed LVM regression was also associated with office systolic BP change over 18 weeks (β = 0.3, p = 0.04).

### Left ventricular and left atrial volumes

LV and LA volumes and derived parameters are given in (Table 2).

**Table 2:** Left ventricular (LV) and left atrial (LA) dimensions and derived parameters determined by CMR imaging before and after 18 weeks’ antihypertensive treatment

Variable	Before treatment	After treatment	P value
LV end-diastolic volume (ml)	130.7 ± 28.0	125.4 ± 25.7	< 0.001
LV end diastolic volume index (ml/m^2^)	64.4 ± 12.0	61.8 ± 10.8	< 0.001
LV end-systolic volume (ml)	45.8 ± 16.6	46.7 ± 15.9	0.04
LV end-systolic volume index (ml/m^2^)	22.6 ± 8.0	23.1 ± 7.7	0.04
LV stroke volume (ml)	84.9 ± 16.5	78.6 ± 14.4	< 0.001
LV ejection fraction (%)	65.6 ± 6.8	63.4 ± 7.1	0.03
LV sphericity index	0.6 ± 0.05	0.6 ± 0.06	0.2
LA volume (ml)	73.3 ± 19.9	68.9 ± 18.5	0.2^a^

Expressed as mean ± standard deviation

^a^Wilcoxon’s signed ranks test

Over 18 weeks, LV end-diastolic volume (LVEDV) and LVEDV index reduced significantly whereas LV end-systolic volume (LVESV) and LVESV index increased significantly following antihypertensive treatment. In accordance with this, stroke volume reduced over the study period, as did LVEF. There was no significant change in LA volume.

When participants prescribed β-blockers were excluded from the analysis, changes in LVESV and LVESV index were no longer significant, though the finding of significant reductions in LVEDV, LVEDV index, LVEF and stroke volume persisted. In addition, sub-group analyses were conducted for each class of medication, with no new significant results found.

### Left ventricular strain before and after antihypertensive treatment

**Fig. 4:**
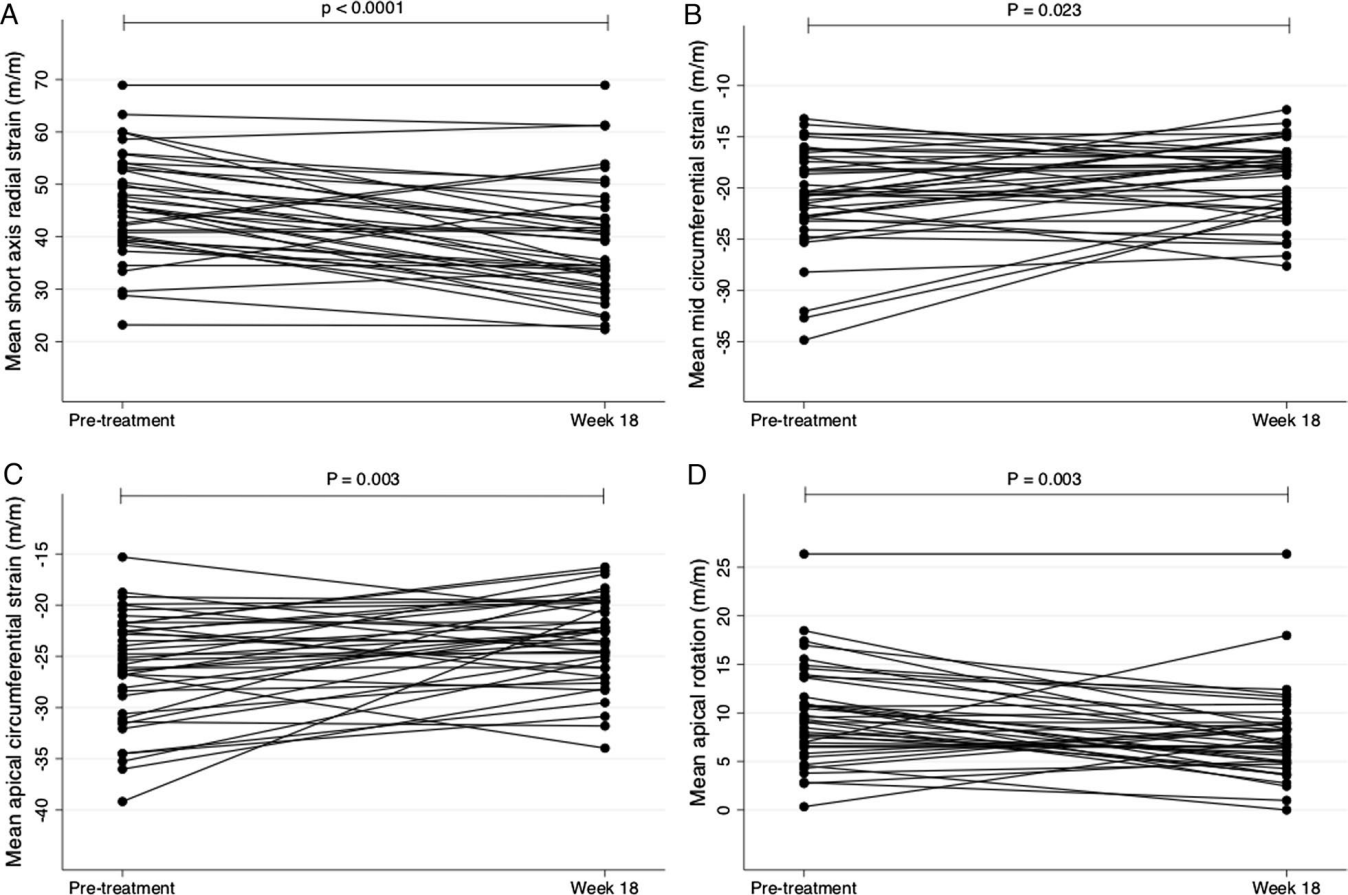
Radial strain (measured in the short axis) (**A**), mid-circumferential strain (**B**), apical circumferential strain (**C**) and apical rotation (**D**) before and after 18 weeks of antihypertensive treatment

Feature tracking analysis of all CMR study participants revealed the reduction in global LVEF and stroke volume following antihypertensive treatment was predominantly linked to a significant reduction in radial strain (measured in the short axis view), a reduction in mid to apical circumferential strain and a reduction in apical rotation (Table 3, Fig. 4). Longitudinal strain trended towards increasing with treatment, whereas torsion tended to decrease with treatment, though these changes were not statistically significant.

**Table 3:** Left ventricular strain before and after 18 weeks’ antihypertensive treatment

Strain parameter (m/m)		Before treatment	After treatment	P value
Radial strain (short axis)				
Endocardial		48.5 ± 18.2	41.7 ± 19.7	< 0.001
Mean		46.1 ± 9.7	39.1 ± 10.9	< 0.001
Radial strain (long axis)				
Endocardial		29.0 ± 9.2	27.4 ± 9.6	0.4
Mean		29.0 ± 9.2	27.4 ± 9.6	0.4
Longitudinal strain				
Endocardial		− 20.3 ± 5.3	− 21.0 ± 6.0	0.4
Mean		− 19.1 ± 4.7	− 19.4 ± 5.4	0.6
Basal circumferential strain				
Endocardial		− 28.7 ± 4.7	− 27.6 ± 4.5	0.1
Mean		− 20.4 ± 3.9	− 19.9 ± 3.4	0.4
Mid circumferential strain				
Endocardial		− 29.9 ± 6.5	− 27.0 ± 5.2	0.003
Mean		− 20.8 ± 4.9	− 19.1 ± 3.7	0.02
Apical circumferential strain				
Endocardial		− 35.6 ± 6.9	− 32.0 ± 5.9	0.001
Mean		− 26.0 ± 5.3	− 23.4 ± 4.2	0.003
Rotation (apical)				
Endocardial		11.9 ± 6.7	9.4 ± 6.0	0.02
Mean		9.8 ± 5.0	7.5 ± 4.5	0.003
Rotation (basal)				
Endocardial		3.9 ± 3.7	3.8 ± 2.9	0.9
Mean		3.1 ± 2.6	2.8 ± 2.0	0.5
Twist				
Endocardial		15.7 ± 7.6	13.2 ± 6.3	0.03
Mean		12.8 ± 5.9	10.4 ± 4.6	0.006
Torsion				
Endocardial		5.4 ± 3.0	4.9 ± 2.8	0.3
Mean		4.4 ± 2.4	3.8 ± 2.0	0.2

Expressed as mean ± standard deviation

Change in mean radial strain measured in the short axis was not significantly associated with change in systolic BP (β = 0.05, p = 0.8) or diastolic BP (β = 0.2, p = 0.2) over the 18-week study period.

### Late gadolinium enhancement in treatment-naïve moderate-severe hypertension

30 participants underwent LGE imaging at baseline, the remaining 11 CMR participants either declining this aspect of the imaging protocol or not receiving contrast due to equipment failure. Seven (23%) were found to have myocardial LGE. The most frequent segment affected was the basal inferoseptum (71%), with the remainder affecting exclusively the basal inferior or basal inferolateral segments. In those with basal inferoseptal or basal inferior LGE, Anderson-Fabry disease was excluded through measurement of serum α-galactosidase.

All LGE was subepicardial in distribution and therefore not indicative of underlying coronary artery disease. There were no significant changes in LGE pattern in any participants after 18 weeks.

## Discussion

This is the first study to examine the effect on myocardial structure and function of a rapid, 18-week, intensive antihypertensive treatment strategy. Using CMR, our study demonstrates that LVH regresses rapidly after only 18 weeks intensive antihypertensive treatment and that this is associated with further, potentially beneficial, alterations in cardiac physiology.

Prior to the current study, the most rapid observed regression of LVH during hypertension treatment was measured at 24 weeks [8] and this study therefore adds credence to the proposal in recent consensus guidelines that benefits can be gained from aiming to control BP in a shorter timeframe than 24 weeks for grade II–III hypertension [27]. Furthermore, the novel use of CMR feature tracking in assessing all directional components of myocardial deformation in a longitudinal study before and after the introduction of antihypertensive agents has for the first time found that radial strain reduces with treatment, whilst also confirming the previously described higher longitudinal strain, lower mid and apical circumferential strain and lower apical torsion in treated hypertension compared with untreated hypertension [8, 10, 15, 16].

The 2018 European Society of Hypertension guidelines for the treatment of hypertension recommend that patients with grade II-III hypertension are treated to target within 3 months in order to improve prognosis [27]. The proposed prognostic benefit for rapid treatment of hypertension is supported by observational evidence from clinical records demonstrating that a delay in achieving target BP is associated with adverse outcomes [28, 29]. Additionally, retrospective analyses of major hypertension trials have revealed that early and effective BP treatment reduces subsequent adverse events with an apparent legacy effect beyond the period of treatment delay [30, 31]. In the present study, a 14% reduction in LVM index was observed, as compared with a 17% reduction in LVM index found in the losartan treatment arm of the LIFE study [32] and the 8% reduction in LVM index detected in a study of intensive antihypertensive treatment delivered over 24 weeks [8]. The rapid reversal of LVH demonstrated by this study may suggest one of the mechanisms by which improved prognosis could be gained and indicate that an appropriately-designed randomised controlled trial should be considered to examine this.

The current study also explores functional changes in the myocardium following antihypertensive treatment, demonstrating that supra-normal LVEF is reduced by treatment, predominantly as a result of a reduction in LVEDV. Although it could be argued that the negative inotropic effect of β-blockade could influence these results, it was noted that the statistically significant observation persists when participants receiving β-blockers at week 18 are excluded from the analysis. LV stroke volume was also found to be lower following antihypertensive treatment in the present study, despite the fact that untreated hypertension has been found to be associated with a lower stroke volume when compared with normotensive control subjects [33]. We propose that this finding is related to the pharmacological effects of antihypertensive agents used within the treatment protocol, with both angiotensin receptor blockers and diuretics likely to confer a reduction in stroke volume.

This study also shows a reduction in radial strain with treatment (when measured in the short axis), a finding which has not previously been demonstrated. This reduction in radial strain is in keeping with the demonstrated reduction in LVEDV with treatment and Starling’s Law, though discrepant with previous cross-sectional data using echocardiographic techniques, which suggested a lower radial strain in untreated hypertension when compared with normotensive controls [10, 11] and subjects with treated hypertension [10]. This difference compared with previous studies may relate to the variance in imaging modalities used to assess radial strain, with the CMR-based technique used in our study known to be more accurate than the echocardiography techniques used in comparable studies. Moreover, the cross-sectional study designs used by previous studies are susceptible to inter-group confounding variables, present to a lesser extent in our longitudinal study design.

Our finding of reduced radial strain following antihypertensive treatment may relate to the concurrent reduction in LV radial thickness also observed, as LVH has previous been demonstrated to be associated with increased radial strain [34]. Alternatively, this finding may be a product of the unique population studied compared with previous investigations (treatment-naïve grade II-III hypertension) or an early response to treatment, which then reverses. To study the possibility of the latter explanation, additional imaging of participants at a later timepoint in their treatment would be informative.

Longitudinal strain tended to increase with treatment of hypertension, which is in keeping with previous cross-sectional and longitudinal studies [8, 10]. In our study, this increase in longitudinal strain did not reach statistical significance, though this is likely to be due to the fact that we found this measurement to be highly variable in untreated hypertension.

We also demonstrated a reduction in circumferential strain in the mid and apical segments in response to antihypertensive treatment. As with previous studies, functional disruption of the heart when exposed to increased afterload appears to affect apical segments to a greater extent than basal segments [10]. This was also the case in the analysis of LV rotation, with increased apical torsion in untreated hypertension compared with treated hypertension in keeping with a previous cross-sectional study [10]. As expected, this translated to increased LV twist in untreated hypertension, though the difference became statistically insignificant when corrected for LV length. This may be due to the reduction in LV length in treated hypertension, as shown by the reduction in LVEDV, which would tend to reduce the torsion measurement.

In addition to myocardial changes with treatment, our protocol aimed to characterise the presence of focal fibrosis within the myocardium in hypertensive heart disease, as determined using LGE. This identified non-ischaemic focal fibrosis in the myocardium of 23% participants, with predilection for affecting the basal infero-septal segment, in agreement with previous observational studies of similar patient groups [35–37].

### Limitations

The observations in this study are based on a broad range of antihypertensive medication, as dictated by the treatment protocol and reported in Table 1. Although a meta-analysis has shown some variability in effect on LVM index between different classes of antihypertensive agents [38], these heterogeneities appear to be relatively small when only the classes of agents recommended in the most recent consensus guideline iterations are considered. It is unknown, however, if there is heterogeneity in the functional response of the myocardium to different antihypertensive agents, though the majority of participants in our study received the standard combination of renin-angiotensin axis blockade and calcium-channel blocker with or without the addition of a thiazide diuretic, ensuring that our results are translatable to clinical patients.

In terms of LGE, this relies on visual determination of the inversion time based on nulling of the “normal” myocardium. If diffuse myocardial fibrosis occurs in hypertension, this would not be visualised if the process homogeneously affects the whole heart and therefore would not have been detected by our CMR protocol, a limitation of the study. However, diffuse fibrosis can be detected using T1 mapping and this has previously been utilised in participants with essential hypertension [36], revealing no significant myocardial fibrosis between the groups of treated hypertensive patients (mean office BP: 152/88 mmHg) versus normotensive controls (mean office BP: 123/74 mmHg). However, this study found a significant degree of diffuse fibrosis in the subgroup of patients with LVH. As LVH regresses with antihypertensive treatment, it can be postulated that this group of patients reflects those with poorly-controlled hypertension. As such, diffuse fibrosis may be a feature of uncontrolled hypertension, which would have not been evident in our study in the absence of T1 mapping. Further investigation for diffuse fibrosis using T1 mapping in grade II/III uncontrolled hypertension may therefore be warranted.

There were 16% of subjects enrolled in the treatment programme who could not undergo CMR testing as a consequence of claustrophobia, which is a higher proportion than would be expected. This may relate to the larger body habitus of participants compared to the population average, particularly when considered alongside the 60cm bore size of the CMR hardware. In addition, the study protocol dictated that CMR examinations were performed after a significant number of microvascular investigations, which may have led to fatigue amongst participants, contributing to aborted examinations.

## Conclusions

The present study demonstrates rapid improvement in LVH as a consequence of implementation of an intensive antihypertensive treatment protocol. These structural changes are accompanied by functional changes in the heart which include a reduction in LVEDV, radial strain, mid and apical circumferential strain and apical rotation.

Given the improved structural and functional parameters seen on CMR following a relatively short period of antihypertensive treatment for newly diagnosed hypertension, early and aggressive treatment of hypertension could plausibly lead to improved clinical outcomes. In the absence of a dedicated randomised clinical trial, these data support a strategy of early blood pressure control, as recommended in the latest European Society of Hypertension guidelines [27].

## Data Availability

The datasets used and/or analysed during the current study are available from the corresponding author on reasonable request.

## Abbreviations

BP: Blood pressure
BSA: Body surface area
CMR: Cardiovascular magnetic resonance
eGFR: Estimated glomerular filtration rate
GCS: Global circumferential strain
GLS: Global longitudinal strain
GRS: Global radial strain
LA: Left atrial
LGE: Late gadolinium enhancement
LV: Left ventricular
LVEDV: Left ventricular end-diastolic volume
LVEF: Left ventricular ejection fraction
LVESV: Left ventricular end-systolic volume
LVH: Left ventricular hypertrophy
LVM: Left ventricular mass
NIHR: National Institute for Health Research
